# Prevalence of anemia and associated factors among adult diabetic patients attending Bale zone hospitals, South-East Ethiopia

**DOI:** 10.1371/journal.pone.0264007

**Published:** 2022-02-18

**Authors:** Damtew Solomon, Kebebe Bekele, Daniel Atlaw, Ayele Mamo, Habtamu Gezahegn, Tadele Regasa, Getahun Negash, Eshetu Nigussie, Demissu Zenbaba, Zinash Teferu, Fikadu Nugusu, Gela Atlie

**Affiliations:** 1 Anatomy Department, Madda Walabu University, Goba, Oromia, Ethiopia; 2 Surgery Department, Madda Walabu University, Goba, Oromia, Ethiopia; 3 Pharmacy Department, Madda Walabu University, Goba, Oromia, Ethiopia; 4 Physiology Department, Madda Walabu University, Goba, Oromia, Ethiopia; 5 Biochemistry Department, Madda Walabu University, Goba, Oromia, Ethiopia; 6 Medical Laboratory Department, Madda Walabu University, Goba, Oromia, Ethiopia; 7 Public Health Department, Madda Walabu University, Goba, Oromia, Ethiopia; 8 Internal Medicine Department, Madda Walabu University, Goba, Oromia, Ethiopia; Indiana University Purdue University at Indianapolis, UNITED STATES

## Abstract

**Background:**

Anemia found in diabetes patients is often unrecognized like many other chronic diseases. The occurrence of anemia is also an additional burden to the micro vascular complications of patients with diabetes. In the selected study structure no published data were found on the prevalence of anemia and associated factors in diabetic patients. Hence, the findings of this study are very fruitful as an input for further studies and after the repetition of similar studies in different frameworks. It is helpful as input for the development of guidelines at diabetes clinics to request the laboratory assessment of hemoglobin as a routine activity.

**Objective:**

This study aimed to assess the prevalence of anemia and its associated factors among diabetic patients who attended Bale zone hospitals.

**Methods:**

A cross-sectional study design was conducted from September 2020–to January 2021 GC among adult diabetic patients who had follow-up at Bale zone hospitals. A total of 238 study participants were determined by single population proportion sample size calculation formula taking prevalence of anemia among adult diabetic patients 19.0%. Systematic random sampling technique was used to select the study participants. Information on demographic and associated factors of anemia in diabetic patients was collected using an interviewer-administered questionnaire. Blood sample collection was performed under aseptic conditions by a licensed medical laboratory professional. Data were entered into EpiData version 3.1, cleaned and exported to statistical package for the social sciences (SPSS) version 25 software tools. Logistic regression was used to assess factors associated with anemia in diabetic patients. P-value less than 0.05 and 95% CI were considered as statistically significant. The odds ratios were reported to indicate the strength of associations. Frequencies, percentages, charts and tables were used to summarize the characteristics of study participants.

**Results:**

In this study anemia among adult diabetic patients is 18.1% (95% CI (13.2, 23.0%). Multivariable logistic regression analysis revealed that the sex of the study participants and the type of diabetes mellitus were found to be statistically significant to associate with anemia. The odds of having anemia among females are nearly three times higher when compared with males (AOR 2.78, 95% CI 1.40–5.52). In addition, the odds of having anemia among adult diabetic patients who had type II diabetes mellitus (AOR 2.18, 95%CI 1.04–4.54) were 2.18 times higher than those who had type I diabetes mellitus patients.

**Conclusion:**

Nearly one out of five adult diabetic patients had anemia. Sex of the patients and the type of diabetes are associated with anemia among adult diabetic patients.

## Introduction

Diabetes mellitus is a group of metabolic disorders characterized by chronic increased blood glucose (hyperglycemia) from different etiologies. Clinical sign and symptoms related to hyperglycemia are: polydipsia (thirst), polyuria (excess urination), polyphagia (sense of hungriness) and sudden weight loss [1]. According to American Diabetic Association (ADA), it is classified as follows: type I, type II, gestational diabetes, and other specific types caused by factors; such as drugs, chemicals, and disease of the pancreas [2].

According to 2016 WHO (World Health Organization) report in 2012, 1.5 million deaths were directly caused by diabetes and 2.2 million deaths by diabetic complications such as chronic kidney diseases and tuberculosis related to hyperglycemia globally [3].

Diabetic patients suffer from micro vascular complications such as, retinopathy, neuropathy, and nephropathy which accounts for 35.7%, 44.6%, and 77%, respectively. From these complications nephropathy has more magnitude (80.6%) among anemic patients than among non-anemic diabetic patients (34.4%) [4].

Anemia is defined as hemoglobin level < 13 g/dl in men and 12g/dl in females [5]. In addition to this, deficiency in the oxygen-carrying capacity of blood caused by a diminished erythrocyte mass or reduction in the hemoglobin concentration of the blood may indicate anemia [6]. It is a condition in which the number of red blood cells (and consequently their oxygen-carrying capacity) is insufficient to meet the body’s physiologic needs. Specific physiologic needs vary based on a person’s age, gender, residential elevation above sea level (altitude), smoking behavior, and different stages of pregnancy [7].

In 2013, 1.93 billion people suffered from anemia worldwide, in which it was abundant in central and western Sub-Saharan countries with a prevalence rate of 45.1% and 43.2% respectively [8]. Diabetic patients are more affected by anemia when compared with non-diabetic patients. Socio—demographic characteristics and glycemic index are statistically correlated with anemia among type II diabetic patients [9]. An increase in evidence suggests that anemia in the diabetic population, whether type I or type II, is a potent and independent predictor of the increased risk for macro-vascular and micro-vascular complications of diabetes [10]. Despite these facts, anemia is unrecognized and largely untreated in patients with diabetes in Ethiopia [11].

Research pertaining facility-based prevalence of anemia among diabetic patients is important to determine if treatment policies need to be adjusted. Thus, the findings of the present study tried to identify diabetic patients having anemia and associated factors that might help in the management of the problem. The results of the study were communicated to Bale zone health bureau that help them to have updated information regarding the prevalence of anemia among diabetic patients. The findings of the study benefitted the study participants involved in the study to know whether they have anemia or not. The result of the study can be also used as input for pooled research analysis that may influence the national policy.

Thus far, no published studies were found in this study framework and it is essential to research the prevalence of anemia among adult DM patients and to implement health interventions including early treatment of anemia among these patients.

## Material and methods

### Study design and area

A hospital-based, cross-sectional study was conducted at Bale zone hospitals from September 2020 to January 2021 GC. Bale zone is situated in the southeast of Ethiopia, 445 km from the capital city of Addis Ababa. The zone has six hospitals namely Robe General Hospital, Dellomena General Hospital, Madda Walabu General Hospital, Ginnir General Hospital, Goro General Hospital and Goba Referral Hospital (the only referral hospital in Bale with a total of 185 clinical staff and 365 administrative workers). These hospitals provide services for about two million people annually in the areas of outpatient and inpatient service, emergency, laboratory, pharmacy, major and minor operations, neonatal intensive care, maternal and child health, ART (Anti-Retroviral Therapy) service, physiotherapy, radiology and dermatology services.

### Source population and exclusion criteria

The source population was adult DM patients residing in the Bale zone and the study population was all patients attending the outpatient’s clinic of the hospitals during the data collection period. Diabetic patients who received anemia correction treatment such as iron and transfusion therapy in the last three months of data collection, those with a recent history of blood loss, pregnant mothers, diabetic patients who were critically ill, in coma, and psychotic patients were excluded from the study.

### Sample size determination and sampling techniques

A total of 238 study participants were determined by single population proportion sample size calculation formula taking prevalence of anemia among adult diabetic patients 19.0% [11], standard normal distribution (Z = 1.96), CI of 95%, absolute precision or tolerable margin of error of 5%. Systematic random sampling was used to select study participants. Sampling frame was prepared by using registration logbook consisted of approximately 500 adult DM patients. We have calculated the K value which was 500/238 = 2.1 and based on this interval 238 study participants were selected by a simple random sampling method.

### Study variables

#### Dependent variable

Anemia

#### Independent variables

➢ Socio demographic characteristics like age, sex, residence, monthly income
➢ History of smoking, chewing khat, Alcohol consumption
➢ Type of diabetic mellitus, weight, height, Body Mass Index (BMI)

### Data collection methods

Data collection was conducted following the COVID-19 protocol and it was collected by three data collectors and one supervisor who were health professionals. Interviewer-administered questionnaire was prepared with an English-language version that was translated into the local language (Afaan Oromo) and back-translated to check consistency in its meaning. Information regarding socio-demographic and associated factors of anemia in diabetic patients was collected using an interviewer-administered questionnaire. Blood sample collection was performed under aseptic conditions by a licensed medical laboratory professional. Diabetic complications and glycemic control data were collected from the patients chart. Anthropometric measurements were conducted as follow:

#### Weight

Weight was measured using electronic digital weight scale by putting the scale on firm flat surface. After participant remove foot wear, heavy clothes, and empty out their pockets for heavy items.

#### Height

Height was measured by portable height measuring board by positioning the board on a firm surface against a wall. After participant remove foot wear, standing feats together facing data collector and eyes are at level of ears. Read the height in centimeters at the exact point to the nearest millimeter.

### Data quality control

Three days of training were given to the data collectors with regard to the overall concept of the study. During training, more emphasis was given to the methods of data collection and inclusion and exclusion requirements. Blood sample processing was performed in accordance with the laboratory’s protocol and properly designed data collection materials were used. The principal investigator checked whether the data collectors had been using the right data collection procedures and techniques. A pretest was performed (using 5% of the total study participants) at Dodola primary hospital to check the consistency of the questionnaire.

### Data processing and analysis

Manual checkup of data for its completeness and clarity, coding, and entry into EpiData version 3.1 and statistical package for the social sciences (SPSS) version 25 software tools was performed. Descriptive analysis of data, bivariable logistic regression (P- statistically significant at p< 0.25 to select variables candidate for multivariable) to determine the association of various factors on the outcome variable and multivariable logistic regression to control confounding effect was performed. P-value less than 0.05 at 95% CI were considered as statistically significant. Frequencies, percentages, charts, and tables were used to summarize the characteristics of study participants.

### Operational definitions

#### Compliance on special advised diet

Those patients who totally rely and feed on diets based on a physician’s advice.

#### Habit of vegetable use per week

Use of any kind of vegetable at least once per day.

#### Habit of fruit use per week

Use of any kind of fruit at least once per day.

#### Khat chewing

Defined as regular chewing of khat (a psychoactive substance) for at least 1 year before we conducted the survey.

#### Current alcohol drinking

Defined as drinking of any amount and type of alcohol within 1 year prior to the data collection.

#### Current smoker

Referred to an adult who had smoked cigarettes within 1 year before the data collection period.

#### Sedentary lifestyle

An individual performing physical activity for “less than 25–30 minutes per day” will be considered to have a sedentary life style.

### Ethical approval and consent to participate

Ethical approval for the study was obtained from Madda Walabu University. Informed written consent to be involved in the research has been obtained from each of the study participant after a clear explanation of the study objectives and potential health and patient data confidentiality risks. Each study participant was informed that all data obtained from them will be kept confidential by using codes and instead of any personal identifiers and is meant only for the purpose of the study. Finally, volunteer study participants were enrolled to provide us required information’s and blood samples for examination.

## Results

### Socio-demographic characteristics of study participants

A total of 238 diabetic patients, 100% response rate, with a mean (±SD) of 44.65 ±17.69 years were involved in the study. One hundred forty four (60.5%) of the study participants were males. The majority of the respondents were married (77.7%) followed by single respondents (16.4%). Out of the 238 total respondents, 102(42.9%) of them had no formal education (Table 1).

**Table 1:** Socio-demographic characteristics of adult DM patients attending Bale zone hospitals, Southeast Ethiopia, 2020 (n = 238).

Variables		Frequency (n = 238)	Percentage (%)
Age of the respondents	18–30	68	28.6
	31–45	62	26.1
	46–60	57	23.9
	>60	51	21.4
Sex of the respondents	Male	144	60.5
	Female	94	39.5
Religion of the respondents	Orthodox	106	44.5
	Muslim	114	47.9
	Protestant	16	6.7
	Other*	2	0.8
Ethnicity of the respondents	Oromo	180	75.6
	Amhara	52	21.8
	Tigre	1	0.4
	Other**	5	2.1
Occupation of the respondent	Student	24	10.1
	Farmer	72	30.3
	Government employee	45	18.9
	Merchant	30	12.6
	House wife	42	17.6
	Daily laborers	25	10.5
Level of education of the respondents	No formal education	49	20.6
	Primary education (1–8)	102	42.9
	Secondary education (9–12)	49	20.6
	College and above	38	16.0
Residence of the respondents	Rural	101	42.4
	Urban	137	57.6
Marital status of the respondents	Single	39	16.4
	Married	185	77.7
	Divorced	6	2.5
	Widowed	8	3.4
Monthly income of the respondents (Ethiopian birr)	<1000	145	60.9
	1000–2000	35	14.7
	2000–4000	23	9.7
	>4000	35	14.7

Other* = (Wakefata, Catholic)

Other** = (Gurage, Sidama).

### Clinical conditions, complications, and comorbidities of the study participants

**Fig 1:**
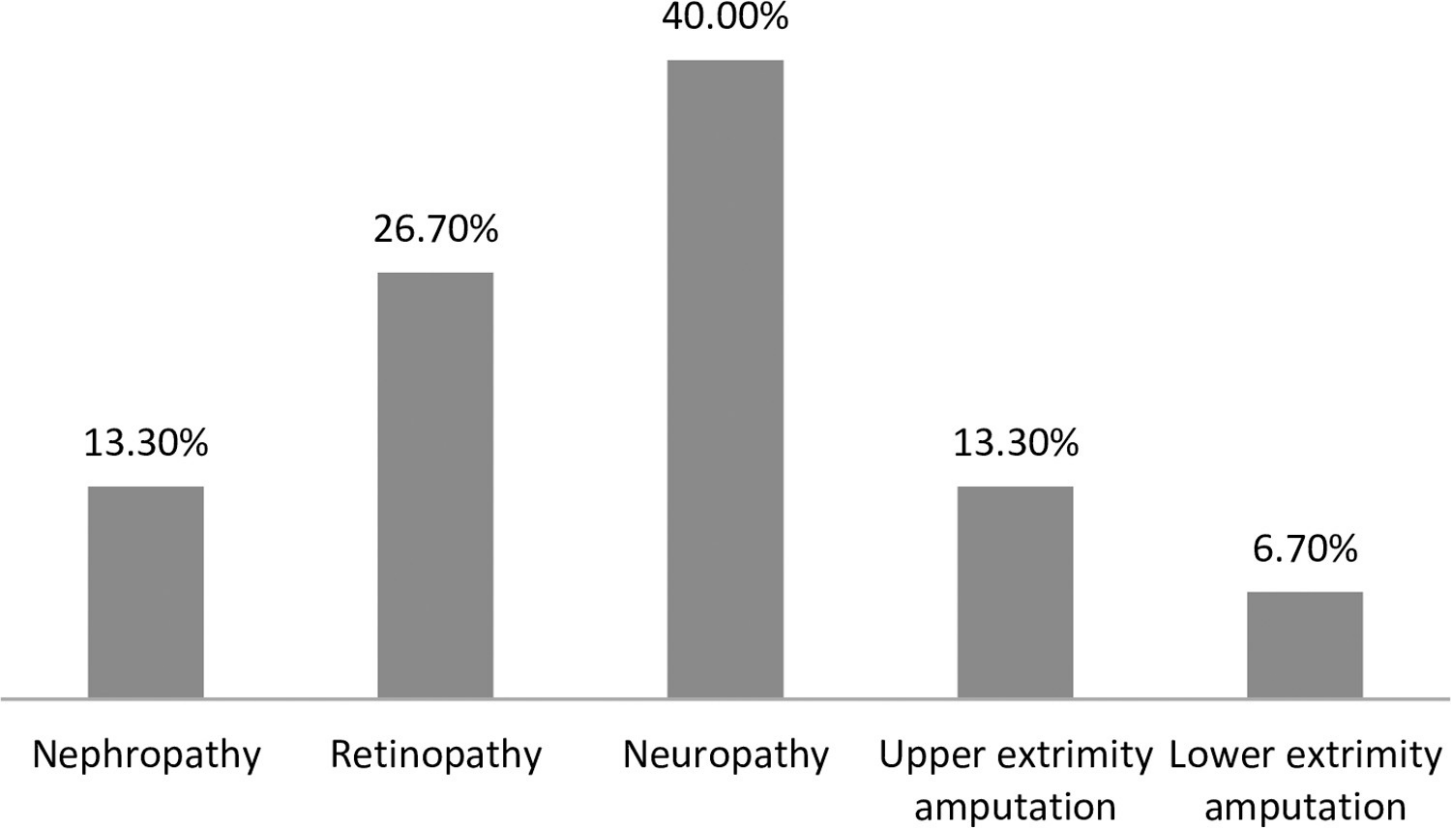
The prevalence of diabetic complications among adult diabetic patients attending Bale zone hospitals from September 2020 to January 2021.

The duration of DM ranged from 1 year to 30 years, with a mean (±SD) of 6.5±5.8 years. Out of the total respondents, 113 (47.5%) were with greater than ten years’ duration of DM followed by 68 (28.6%), who had less than five years duration. Every study participant was taking medications for the management of diabetes. The majority of the respondents, 147 (61.8%) were taking injectable medication. One hundred fifty two (63.9%) had normal body mass index at the time of data collection (18.5–24.9 kg/m^2^) and 55 (23.1%) of them were overweight (BMI (25–29.9 kg/m^2^). Fifteen (6.3%) of the study participants were presented with records of diabetes-related micro vascular complications. Neuropathy 6 (40%) was the most prevalent complication followed by diabetic retinopathy 4(26.7%) and nephropathy 2 (13.3%). Upper extremity 2(13.3%) and lower extremity 1(6.7%) amputations for unrecorded reason were also identified from their records (Fig 1). Sixty eight (28.6%) of the study participants were hypertensive. Sixty (25.2%) and sixty eight (28.6%) of the study participants had SBP of ≥ 130 mmHg and DBP of ≥80 mmHg, respectively. Eighteen of the study participants had asthma. The average of four consecutive fasting blood glucose levels (FBG) ranged from 78 to 478 mg/dl with a mean (± SD) of 208.58 ±72.95 mg/dl. The majority of the study participants, 209 (87.8%), were presented with poor glycemic control (Table 2).

**Table 2:** Clinical characteristics of adult DM patients attending Bale zone hospitals, Southeast Ethiopia, 2020 (n = 238).

Type of diabetes, n (%)	Type I	103(43.3)
	Type II	135(56.7)
Duration of diabetes, n (%)	< 5 years	68(28.6)
	5–10 years	57(23.9)
	> 10 years	113(47.5)
Hypertension, by Systolic BP n (%)	60 (25.2)	
Hypertension, by Diastolic BP n (%)	68 (28.6)	
Stages of Hypertension by Systolic BP, n (%)	Stage I	35(14.7)
	Stage II	22(9.2)
	Severe Hypertension	3(1.3)
Stages of Hypertension by Diastolic BP, n (%)	Stage I	38(16)
	Stage II	28(11.8)
	Severe Hypertension	2(0.8)
Body Mass Index, n (%)	<18.5	20(8.4)
	18.5–24.9	152(63.9)
	25–29.9	55(23.1)
	> = 30	11(4.6)
Nature of glycemic control, n (%)	Good glycemic control	29(12.2)
	Poor glycemic control	209(87.8)
Family history of DM, n (%)	Yes	41(17.2)
	No	197 (82.8)

### Lifestyle and nutritional status of the study participants

Based on the findings of the present study, only 3 (1.3%) of the participants had a history of cigarette smoking at least once in their lifetime. In addition, 10(4.2%) of the study participants had a history of alcohol use and 8(3.4%) of study participants used to chew khat. Moreover, 177(74.4%) of the respondents were not engaged in even moderate physical activities such as riding a bicycle, or walking for 30 min per day. When their nutritional status was considered, nearly one fourth 55(23.1%) of the study participants were overweight and only 11(4.6%) of the study participants were obese. The rest of the variables are shown in (Table 3).

**Table 3:** Lifestyle and nutritional status of adult DM patients at Bale zone hospitals from September 2020 to January 2021 (n = 238).

Habit of vegetable use per week, n (%)	<3days	126 (52.9)
	3-5days	68 (28.6)
	>5days	44 (18.5)
Habit of fruit use per week, n (%)	<3days	218 (91.6)
	3-5days	15 (6.3)
	>5days	5 (2.1)
Number of sitting hours per day, n (%)	<5hours	126 (52.9)
	5-8hours	76 (31.9)
	>8hours	36 (15.1)
Type of oil used for meal preparation in the home, n (%)	Packed and bottled oil	189(79.4)
	Butter	7(2.9)
	Vegetable oil	34(14.3)
	Other	1(0.4)
	None used	3 (1.3)
	Don’t know	4(1.7)
History of traditional medicine use in past one year, n (%)	Yes	29 (12.2)
	No	209 (87.8)
Choice of food based on health professional advice, n (%)	Yes	222 (93.3)
	No	16 (6.7)
Performing work that involves vigorous activity, n (%)	Yes	42 (17.6)
	No	196 (82.4)
Simple walk for about 30 minutes per day, n (%)	Yes	61 (25.6)
	No	177 (74.4)

### Prevalence of anemia among DM patients

**Fig 2:**
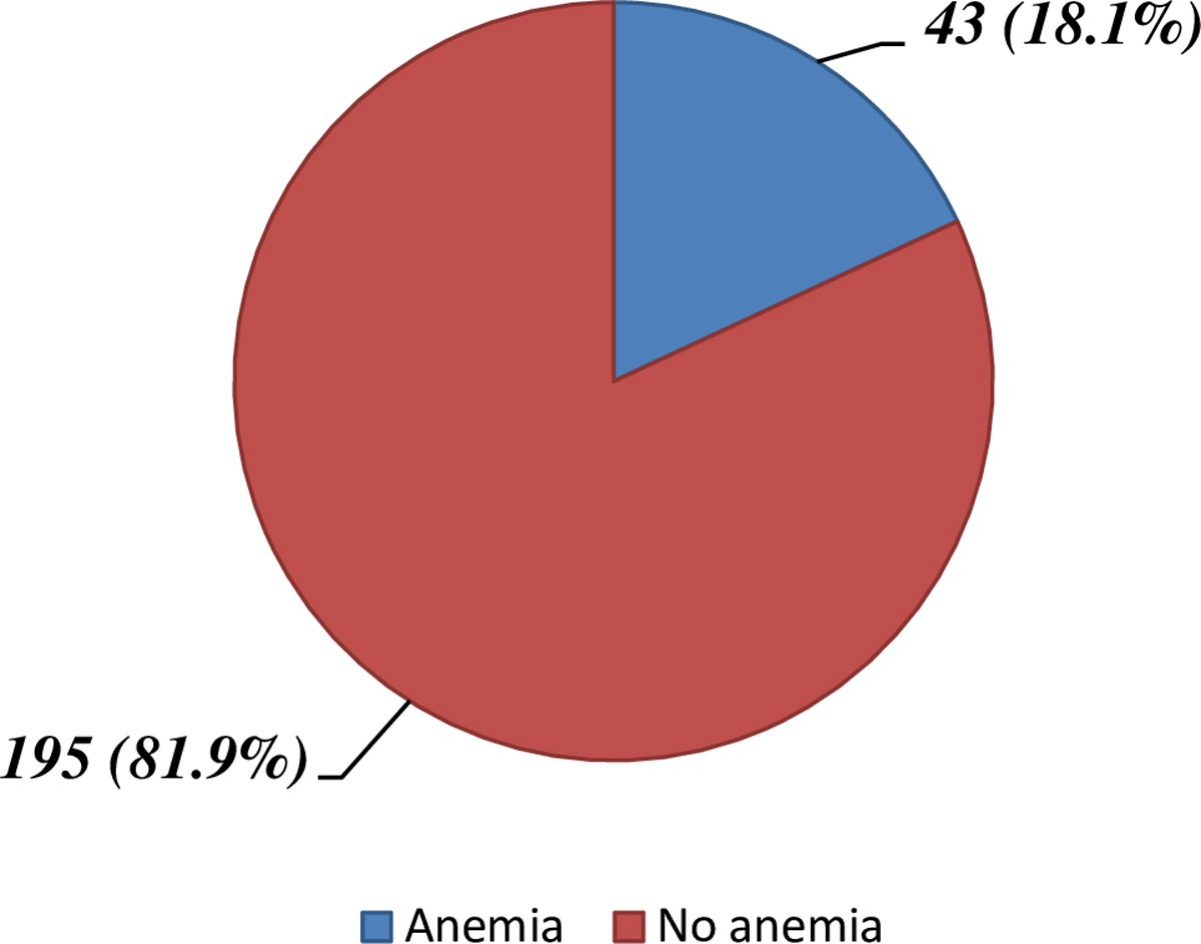
Prevalence of anemia among adult DM patients attending Bale zone hospitals South-east Ethiopia, from September 2020 to January 2021.

**Fig 3:**
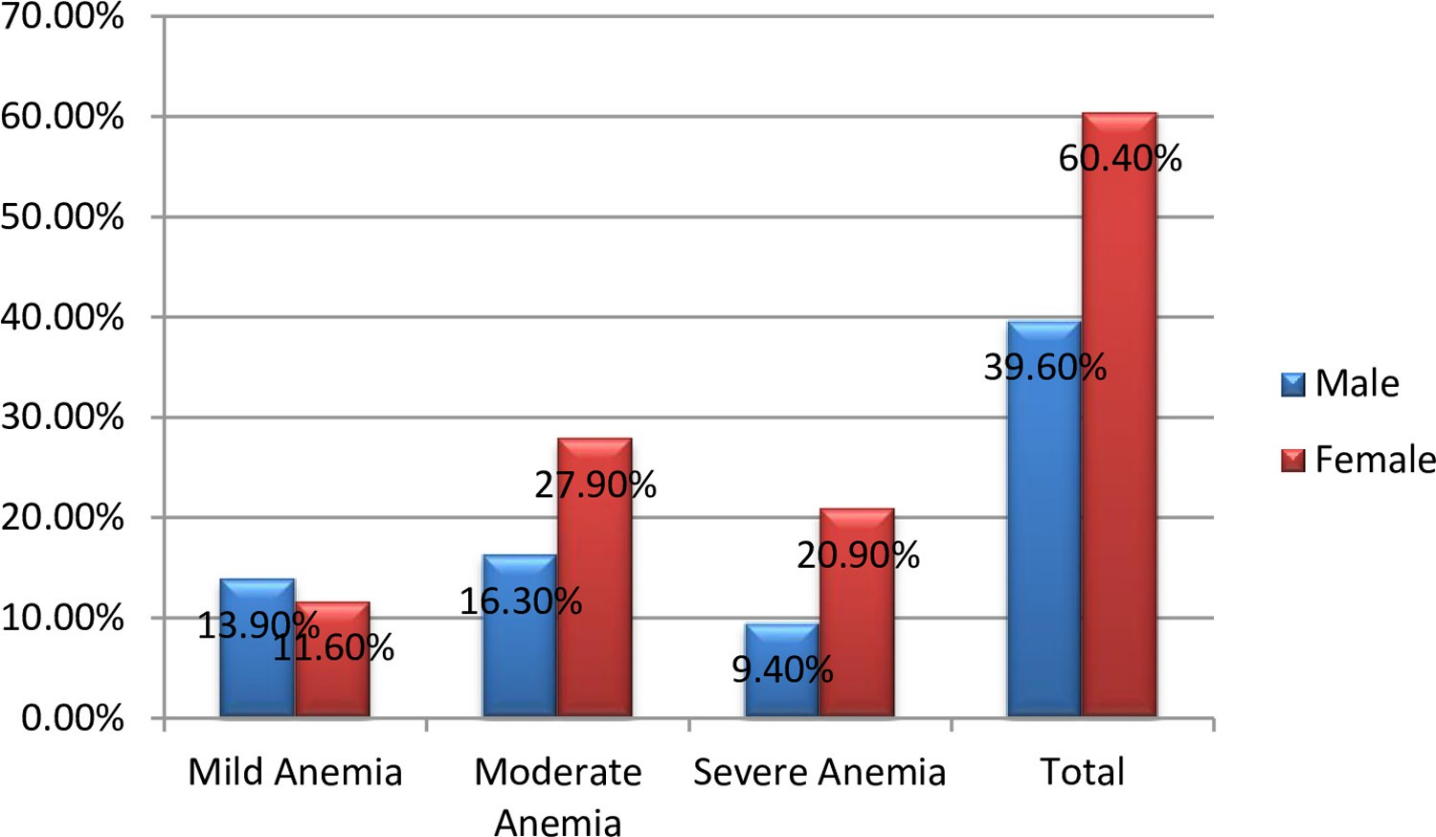
Prevalence and degree of anemia among adult DM patients attending Bale zone hospitals from September 2020 to January 2021.

Hemoglobin level of the study participants was, from 4.5 g/dl to 19.9 g/dl, with a mean (± SD) of 14.19 ± 2.78 g/dl. Out of the 238 total study participants, 43 (18.1%, 95% CI (13.2, 23.0%). of them had anemia with 17 (19.01%) in males, and 26 (21.1%) in females, Fig 2. Out of the anemic DM patients, 11 (25.6%) and 19 (44.2%) had mild and moderate anemia, respectively, Fig 3. From these anemic patients, none of them was ever screened for anemia before. Among the anemic patients, approximately 81.4% of the study participants had normocytic hypochromic type of anemia followed by macrocytic hypochromic type accounting 11.6% (Table 4).

**Table 4:** Shows the prevalence of anemia and its type among adult DM patients attending Bale zone hospitals South-east Ethiopia, from September 2020 to January 2021.

Variables		Frequency (n = 238)	Percentage (%)
Anemia status	Absent	195	81.9
	Present	43	18.1
Types of anemia	normocytic hypochromic	35	81.4
	microcytic hypochromic	3	7.0
	macrocytic hypochromic	5	11.6

### Factors associated with anemia among DM patients

#### Bivariate analysis of variables

Bivariable analysis was performed observe the association of each selected factor (independent variable) with the occurrence of anemia (dependent variable). Bivariable analysis of anemia demonstrated that age, sex, occupational status, level of education, marital status, having co-existing disease, number of hours patient sit per day, complying on special prescribed diet, food choice, the amount of vegetables consumed per week, BMI, and type of diabetes mellitus, are variables candidate for the next model, the multivariable logistic regression model. Accordingly, DM patients who had type II DM were nearly two times (COR = 2.26, 95% CI (1.09–4.66) at a greater risk to be anemic than type I diabetes mellitus patients (Table 5). In the table below, the candidate variables for multivariable analysis are summarized.

**Table 5:** Results of bivariate logistic regression of selected variables in relation to anemia among adult diabetes mellitus patients attending Bale zone hospitals from September 2020 to January 2021.

Variables	Anemia		COR (95% CI) and P- value	
	Yes	No	COR (95% CI)	P- value
**Age groups**				
18–30	6	62	1	
31–45	14	48	3.01 (1.08–8.42)*	0.035
46–60	10	47	2.20 (0.75–6.48)*	0.153
>60	13	38	3.53 (1.24–10.08)*	0.018
**Sex**				
Male	17	127	1	
Female	26	68	2.86 (1.45–5.63)*	0.02
**Educational status**				
No formal education	15	34	2.35 (0.81–6.81)*	0.115
Primary education(1–8)	18	84	1.14(0.42–3.14)	0.795
Secondary education (9–12)	4	45	0.47 (0.12–1.82)	0.276
College and above	6	32	1	
**Occupation**				
Student	1	23	1	
Farmer	17	55	7.11 (0.89–56.6)*	0.064
Government employee	5	40	2.87 (0.32–26.14)	0.348
Merchant	2	28	1.64 (0.14–19.29)	0.693
House wife	12	30	9.2 (1.11–75.97)*	0.039
Other	6	19	7.2 (0.80–65.70)*	0.078
**Marital status**				
Single	5	34	1	
Married	32	153	1.42 (0.52–3.92)	0.496
Divorced	2	4	3.4 (0.49–23.65)*	0.216
Widowed	4	4	6.8 (1.27–36.26)*	0.025
**Presence of co-existing disease**				
Yes	18	60	1.63 (0.82–3.19)*	0.163
No	25	135	1	
**Number of hours sitting/day**				
<5hours/day	25	101	1	
5-8hours/day	10	66	0.6 (0.276–1.35)*	0.227
>8hours/day	8	28	1.15(0.47–2.84)	0.755
**Compliance on special advised diet for DM patients**				
Yes	20	23	1.7 (0.87–3.32)*	0.12
No	66	129	1	
**Availability of food choice in the house**				
Yes	37	185	1	
No	6	10	3 (1.03–8.76)*	0.045
**Number of days the patient consumes any type of vegetables per week (at least once per day)**				
<3days/week	3	41	3.73 (1.07–12.97)*	0.039
3-5days/week	13	55	3.23 (0.86–12.08)*	0.081
>5days/week	27	99	1	
**Body Mass Index**				
<18.5 (underweight)	2	18	1	
18.5–24.9(normal)	31	121	2.3 (0.51–10.47)	0.279
25–29.9(overweight)	7	48	1.3 (0.25–6.92)	0.748
≥30(obese)	3	8	3.4 (0.47–24.29)*	0.227
**Type of Diabetes Mellitus**				
Type I	12	91	1	
Type II	31	104	2.26 (1.09–4.66)*	0.027

*Statistically significant at p< 0.25, 1 = the reference group. COR, crude odd ratio.

#### Multivariate analysis of variables

Factors that showed a p-value ≤ 0.25 in the bivariable analysis were considered as candidates to the multivariate logistic regression model to identify the most significant determinant of anemia.

The multivariable logistic regression analysis which was derived from binary logistic regression analysis revealed that sex of the study participants and the type of DM were found to be statistically significant to associate with anemia among adult diabetes patients. In the table below (Table 6), variables that showed significant association in multivariable analysis are summarized.

**Table 6:** Findings of binary and multivariable logistic regression analysis of anemic status among adult DM patients attending Bale zone hospitals from September 2020 to January 2021 (n = 238).

Variables		Anemia		95% CI	
		Yes	No	COR	AOR
Sex of the study participants	Male			1	1
	Female			2.86 (1.45–5.63)	2.78 (1.4–5.52)*
Type of DM	Type I	12	91	1	1
	Type II	31	104	2.26 (1.09–4.66)	2.18 (1.04–4.54)*

*Statistically significant at p< 0.05, 1 = the reference group. COR, crude odd ratio; AOR, adjusted odd ratio.

## Discussion

In this hospital-based cross-sectional study, the prevalence of anemia and its associated factors among adult DM patients attending Bale zone hospitals South-east Ethiopia has been assessed.

According to the findings of the present study, the prevalence of anemia among adult diabetes mellitus patients is 18.1% (95% CI (13.2,23.0%), which revealed that nearly one out of five adult diabetes mellitus patients had anemia. This prevalence is in accordance with the studies conducted in different parts of Ethiopia [11,12]. However, the prevalence of anemia in our study is lower when compared with findings of the study conducted among patients attending public hospitals in the Harari region of Ethiopia [13], which was 34.8% and the over representation might be because of the inclusion of only type II adult DM patients in the study. In addition to this, greatly different life styles of the two populations also matters. Similarly, results of the study conducted among adult DM patients at Debre Tabor General Hospital demonstrated a higher prevalence of anemia29.8% [14], when compared with findings of the present study. The findings from a study in Pakistan showed a significantly higher prevalence of anemia among type II DM patients [15], which might be caused by a difference in the instrument used to measure the level of hemoglobin. The afore mentioned study used HbA1c to measure hemoglobin level. In another way, the mismatch can be explained by the composition of our study subjects. In this study, the study subjects were both type I and type II DM patients, whereas the mentioned study was conducted only in type II diabetic patients. The author of the above study also tried to explain the possible reason for the increment of anemia that could be caused by poor nutrition, poverty, lack of awareness and illiteracy, as the study was performed in Karachi, Pakistan.

Past studies, [16,17], reported a higher prevalence of anemia in diabetic males, 43 (68.25%) than in diabetic females 20 (31.74%). In contrast, our result indicates that females have nearly three times higher chance of developing anemia when compared with males. Higher rate of anemia in females can be because of monthly physiological blood loss in the form of menstruation and the culture of food eating in this study framework where females fed the rest of family members before having food for themselves. This result is comparable with findings from different studies [15,18,19]. A report from the University of Benin Teaching Hospital stated that there is no significant difference in the risk of anemia between males and females [20], which contradict our findings. These differences can be attributed to the small sample size they have used only 72 type-II diabetic patients were selected for the study.

Even though the presence of anemia is not associated with the duration of diabetes in our study, which is inconsistent with findings of a previous study in the country [17,21], adult patients with type II DM have a higher chance nearly two times, of developing anemia when compared with type I DM patients. This can be explained by the fact that patients with type II DM pass through a period of pre-diabetes and may experience renal impairment at the time of diagnosis, which exposes patients to a higher risk of anemia. Similar to our findings, it has been also suggested by other studies that anemia is related to type II DM patients [17]. Similar findings of study conducted in Northeast Ethiopia demonstrated that having type II DM is nearly 2.4 times risky in developing anemia, when compared with those who have type I DM [22].

## Limitations of the study

This study has some limitations, it would have been more conclusive if it used a comparison group and assessed the level of their blood glucose level using hemoglobin A1c. Because it is a cross-sectional study, it will not show the temporal relation between the independent and dependent variables. There is also a possibility of recall bias as the questionnaire for food consumption was based on recall knowledge. Hemoglobin level was not adjusted for altitude and the level of glycemic status was assessed by fasting blood glucose rather than HbA1c.

## Conclusions

Nearly one out of five DM patients had anemia. Most of the anemic patients had a mild type of anemia. Morphologically, the predominant type of anemia was normocytic normochromic anemia. Sex and type of DM were significantly associated. The findings of our study suggest the necessity for incorporating routine screening for anemia in all DM patients mainly for patients with these identified risk factors to facilitate early detection and management of anemia among DM patients.

## Supporting information

S1 File SPSS Data 1: Anemia spss data.sav 1. (SAV)

S2 File Questionnaire-English Version 1: Anemi 1. (DOCX)

S3 File Questionnaire-Afaan Oromo Version 2. (DOCX)

## Abbreviations

ADA: American Diabetic Association
AOR: Adjusted Odds Ratio
ART: Anti-retroviral therapy
BMI: Body Mass Index
CI: Confidence Interval
COR: Crude Odds Ratio
DBP: Diastolic Blood Pressure
DM: Diabetes Mellitus
FBG: Fasting Blood Glucose
FBS: Fasting Blood Sugar
GRH: Goba Referral Hospital
HbA1c: Hemoglobin A1c
MWU: Madda Walabu University
SBP: Systolic Blood Pressure
SPSS: Statistical Package for the Social Sciences
WHO: World Health Organization
