# Supplementary material for: Prevalence of anemia and associated factors among adult diabetic patients attending Bale zone hospitals, South-East Ethiopia
Source: PLoS One. 2022 Feb 18;17(2):e0264007. doi: 10.1371/journal.pone.0264007 (PMC8856574; doi:10.1371/journal.pone.0264007)
Supplement: S2 File — (DOCX) [file pone.0264007.s002.docx]

# Annex I: Questioners

Hello. My name is _______and I am data collector of the study conducted by Damtew et al., Madda Walabu University academic staffs and researchers. Conducting this research entitled “Prevalence of anemia and associated factors among adult diabetic patients attending Bale zone hospitals, South-east Ethiopia*”*. We would very much appreciate your participation in this survey. The information you provide will help us to determine prevalence and factors associated with anemia. The interview takes between 20-30 minutes to complete. As part of the study, we would first like to ask you socio demographic and take *anthropometric* and *BP* measures and take blood sample. Whatever, information you provide will be kept strictly confidential, and will not be shared with anyone other than members of our research team. Participation in this survey is voluntary, and if we should come to any question you don't want to answer, just let me know and I will go on to the next question; or you can stop the interview at any time. However, we hope you will participate in the survey since your views are important.

**At this time, do you want to ask me anything about the survey?**

**May I begin the interview now?**

**Signature of interviewer: -------------------- Date: ----------/--------/--------**

1. RESPONDENT AGREES TO BE INTERVIEWED🡪 interview
2. RESPONDENT DOES NOT AGREE TO BE INTERVIEWED🡪 end

**For more information and questions here is the contact address of principal investigator.**

**Damtew Solomon**

**Tel: +251920023076**

**E-mail:** [**fraolsolomon675@gmail.com**](mailto:fraolsolomon675@gmail.com)

## Consent form

I_____________________ am informed on study to be conducted by Damtew et al., Madda Walabu University academic staffs and researchers, “Prevalence of anemia and associated factors among adult diabetic patients attending Bale zone hospitals, South-east Ethiopia*”*. participation in this study is voluntary, no obligation to answer any questioner there is no harm by not answering the questions and no special benefit by answering the question and also the interview will take 20- 30 minutes. I heard all the information mentioned above and willing to participate in the interview.

**Name of interviewer_________ Signature________**  (Signature of interviewer certifying that respondent has given informed consent verbally)

| S.No | Questions | Response | Remark |
| --- | --- | --- | --- |
| 101 | How old are you? (age of respondent) | ---------------------year |  |
| 102 | What is your sex? | 1.Male  2.Female |  |
| 103 | What is your religion? | 1.Muslim  2.Orthodox  3.Protestant  4.Other (specify)----- |  |
| 104 | What is your ethnicity? | 1.Oromo  2.Amhara  3.Tigre  4.Other (specify)------- |  |
| 105 | What is your occupation? | 1. Student 2. Farmer 3. G- employee 4. Merchant 5. House wife 6. Other (specify) |  |
| 106 | What is your level of education? | 1. Illiterate 2. Able to read and write 3. Primary education (1-8) 4. Secondary education (9-10) 5. Preparatory education (11-12) 6. College and above |  |
| 107 | Where is your residence? (Place of residence) | 1. Rural 2. Urban |  |
| 108 | What is your marital status? | 1. Single 2. Married 3. Divorced 4. Widowed |  |
| 109 | What is your monthly income? | -------- |  |
| 110 | Do you smoke cigarrate? | 1.No  2.Some times  3. Always |  |
| 111 | Do you chew khat? | 1. No 2. Some times 3. Always |  |
| 112 | Do you drink alcohol? | 1.No  2. Some times  3.Always |  |
| 113 | Have you taken any drug for anemia before 3 months? | 1.Yes  2. No |  |
| 114 | Do you use currently antibiotic for any illness? | 1. YES 2. No |  |
| 115 | Do you have any co-existing disease? | 1. Yes 2. No |  |
| **Physical activity** | | | |
| 116 | How much time do you usually spend sitting or reclining on a typical day | Day---------hours-------- |  |
| 117 | Does your work involve vigorous-intensity activity that causes large increases in breathing or heart rate like [carrying or lifting heavy loads, digging or construction work] for at least 10 minutes continuously? | 1. Yes 2. No |  |
| 118 | In a typical week, on how many days do you do vigorous-intensity activities as part of your work? | No of days--------- |  |
| 119 | In a typical week, on how many days do you do moderate-intensity activities as part of your work? | No of days------------- |  |
| 120 | Do you walk or use a bicycle (pedal cycle) for at least 10 minutes continuously to get to and from places? | 1.Yes  2.No |  |
| 121 | If yes for Q No 114, In a typical week, on how many days do you walk or bicycle for at least 10 minutes continuously to get to and from places? | No of days----- |  |
| **History of DM** | | | |
| 122 | In your family, is there anyone who has DM? (Family history of DM) | 1.Yes  2. No |  |
| 123 | What type of DM you have? (Type of DM? | 1. Type-I 2. Type-II |  |
| 124 | For how long you live with DM? (Duration with DM) | ----- |  |
| 125 | What type of treatment you take? (Type of treatment) | 1. Injectable (lente insulin) 2. Pills (metformin) |  |
| 126 | Do you take your medication as per schedule always? (adherence to medication) | - - - 1. Yes       2. No |  |
| 127 | Do you use home glucose monitoring machine? | 1.Yes  2. No |  |
| 128 | Do you have other diabetic complication? | 1.Yes  2. No |  |
| 129 | If yes for Q No 128 specify the complication | ----------------- |  |
|  | Is there any special prescribed diet for you? | 1.Yes  2.No |  |
| 130 | Do you choose your food based on the advice of Doctor? | 1. Yes 2. No |  |
| 131 | Are you currently taking any herbal or traditional remedy for your diabetes? | 1. Yes 2. No |  |
| **Diet** | | | |
| 132 | In a typical week, on how many days do you eat fruit? | No of days------ |  |
| 133 | In a typical week, on how many days do you eat vegetables? | No of days------ |  |
| 134 | What type of oil or fat is most often used for meal preparation in your household? | 1. Vegetable oil 2. Butter or ghee 3. Margarine 4. None in particular 5. None used 6. Don’t know   Other: _________ |  |
| 135 | On average, how many meals per week do you eat that were not prepared at a home? By meal, I mean breakfast, lunch and dinner. | Number------- |  |

## Laboratory analysis report format

| S. No | Name of laboratory test | Result | Remark |
| --- | --- | --- | --- |
| 1. | Hg |  |  |
| 2. | Fasting blood sugar (FBS) |  |  |
| 3. | Blood pressure |  |  |
| 4. | Anemia status | 1.Present  2.Absent |  |
| 5. | Type of anemia | 1.Microcytic hypochromic  2.Normocytic hypochromic  3.Macrocytic |  |
| Anthropometric measurements report format | | | |

| 1. | Height |  |  |
| --- | --- | --- | --- |
| 2. | Weight |  |  |
| 3. | BMI |  |  |

# 
