# Supplementary material for: Prevalence of anemia and associated factors among adult diabetic patients attending Bale zone hospitals, South-East Ethiopia
Source: PLoS One. 2022 Feb 18;17(2):e0264007. doi: 10.1371/journal.pone.0264007 (PMC8856574; doi:10.1371/journal.pone.0264007)
Supplement: S3 File — (DOCX) [file pone.0264007.s003.docx]

Maxxantu I: Gaaffilee

Ashamaa!! Maqaan koo ___________ dha. Ani qorannoo obbo Yohaannisii fi barsiistota yuunivarsiitii Madda walaabuu birootiin hojjetamuuf odeeffannoo kan sassaabuu dha. Qorannoon raawwatamu kunis mata dureen isaa “hammaa fi wantoota hanqina dhiigaa fidaniin wal qabatan ga’eessota Hospitaalota goodina Baalee fi baha Baalee, kibba baha, Itiyoophiyaa” kan jedhuudha. Qorannoo kana irratti hirmaachuu keetiif baay’ee itti gammadna. Odeeffannoon ati nuuf kennitu hammaa fi wantoota furdina garmalee akkasumas dhibee dhiibbaa dhiigaa wajjin walqabatan safaruuf nu gargaara. Gaaffii fi deebiin kun daqiiqaa 20-30tti ni xumurama. Akka qaama qorannoo kanaatti jalqaba irratti waa’ee hawaaasummaa fi haala uummataa si gaafanna; akkasumas ulfaaatina qaamaa, dheerina fi hamma dhiibbaa dhiigaa kee safarra. Odeeffannoon ati nuuf kennitu kamiiyyuu iccitiidhaan qabama, qaama biraatiifis dabarfamee hin kennamu. Hirmaannaan qorannoo kanaa fedha irratti kan hundaa’ee dha; gaaffiin deebii itti kennuu hin barbaanne yoo jiraate gara gaaffii irra darbuu ni dandeessa yookiin gaffii fi deebicha yeroo kamittuu dhaabuu ni dandeessa. Ta’us, ilaalchi kee barbaachisaa waan ta’eef qorannoo kana irratti akka hirmaattu abdii qabna

**Yeroo kanatti waa’ee qorannichaa ilaalchisee wanti na gaafattu jiraa?**

**Gaaffii fi deebii eegaluu dandeenyaa?**

**1. Yoo hirmaataan sun tole jedhe --🡪 gaaffii fi deebii geggeessi**

**2. Yoo hirmaataan sun dide ---🡪 galateeffadhuu dhaabi**

**Mallattoo nama gaaffii fi deebii geggeessee: -------------------- guyyaa: ----------/--------/--------**

**Odeeffannoo dabalataa fi gaaffiin yoo jiraate teessoo armaan gadiitiin qorataa olaanaa qorranichaa argachuu dandeessu.**

**Daamxaw Salamoon**

**bilbila: +251920023076**

**imeelii:**fraolsolomon675@gmail.com

**Uunka walii galtee**

ani ___________ waa’ee qorannoo obbo Daamxaw fi barsiistota yuunivarsiitii Madda walaabuutiin mata duree “hammaa fi wantoota hanqina dhiigaa fidaniin wal qabatan ga’eessota Hospitaalota goodina Baalee fi baha Baalee, kibba baha, Itiyoophiyaa” jedhu irratti hojjetamu hubadheen jira. Hirmaannaan qorannoo kanaa fedha irratti kan hundaa’ee dha; gaaffii kamiiyyuu deebisuuf dirqamni hin jiru, deebisuu dhabuunis miidhaa hin qabu akkasumas gaaffii deebisuudhaan bu’aan addaa argamu hin jiru. Akkasumas gaaffii fi deebichi daqiiqaa 20-30 fudhata. Odeeffannoowwan armaan oliitti tarreeffaman hundumaa hubachuudhaan qorannicha irratti hirmaachuuf hayyamammaa dha (fedhiin qaba)

**maqaa nama gaaffii fi deebii gaggeessee_________ mallattoo________**

(**mallattoon nama odeeffannoo funaanuu hirmaataan qorannichaa hirmaatichi odeeffanoo kennuuf waliigaluu isaa mirkaneessa**)

**Odeeffannoo waliigalaa** Lakkoofsa hirmaataa qorannichaa: ____________

Gaaffiilee kanatti aansanii jiraniif deebii sirrii ta’e itti marsuu ykn barreessuudhaan guuti

**Kutaa 1: Gaaffiilee haala hawaasummaan walqabatan**

| **Lakk** | **GAAFII** | **DEEBII** | **DARBI** |
| --- | --- | --- | --- |
| 101 | Umrii ( waggaa guutuudhan) | ___________ waggaa |  |
| 102 | Saala (***Hin gaafatin, akkasumaan ilaaluun guuti***) | 1. Dhiira 2. Dhalaa |  |
| 103 | Amantaan kee maaliidha? | 1. Muslima 2. Ortoodoksii 3. Piroteestantii   Kan biroo ________________ |  |
| 104 | Sabni kee maaliidha? | 1. Oromoo 2. Amaara 3. Tigree 4. Kanbiraa _________________ |  |
| 105 | Hojiin kee amma maali? | 1. Barataa 2. Qote bulaa 3. Hojjeetaa mootummaa 4. Daldalaa 5. Haadha Manaa 6. Kan biraa------- |  |
| 106 | Sadarkaa barnoota | 1. Kan baressu fi dubbisuu hin dandeenye 2. Bareessu fi dubbisuu kan danda’u 3. Sadarkaa 1ffaa (1-8) 4. Sadarkaa 2ffaa (9-10) 5. Qophaa’ina (11-12) 6. Kolleejjii fi isaa ol |  |
| 107 | Iddoo jireenya | 1. Magaalaa 2. Baadiyyaa |  |
|  |  |  |  |
| 108 | Haala gaa’ilaa | 1. Kan hinfuunee/hin heerumne 2. Kan fuudhe/kan heerumte 3. Kan waal hiikan 4. Kan abbaa warra hin qabne   Jalaa du’e/duute |  |
| 109 | Galii ji’aan | -------------- |  |
| 110 | Sigaaraa ni aarsita | 1. Lakki 2. Yerootokko tokko 3. Yeroo hunda |  |
| 111 | Caatii ni nyaatta? | 1. Lakki 2. Yerootokko tokko 3. Yeroo hunda |  |
| 112 | Alkooli ni dhugda? | 1. Lakki 2. Yerootokko tokko 3. Yeroo hunda |  |
| 113 | Hanqina dhiigaaf ji’oota 3dura qoricha fudhattee? | 1. Eeyyen 2. Lakki |  |
| 114 | Amma qoricha fudhachaa jirta? | 1. Eeyyen 2. Lakki |  |
| 115 | Dhibee dabalata qabdu? | 1. Eeyyen 2. Lakki |  |
|  | **Sosochii qaamaa** |  |  |
| 116 | Torbanitti guyyaa meeqaaf sochiilee qaamaa ulfaataa armaan olitti ibsaman hojii keessan irratti sin qunnama? | Guyyaan-sa’aati---- |  |
| 117 | Hojiin keessan sochii qaamaa ulfaataa kanneen dhahannaa laphee ykn hargansuu baay’ee dabalan ni dabalataa? Fkn: yoo xiqqaate daqiiqaa 10 fi isaa oliif walitti aansuudhaan ba’aa ulfaataa baachuu, qotuu, hojii ijaarsa adda addaa hojjechuu. | 1. Eeyyen 2. Lakki |  |
| 118 | Torbanitti guyyaa meeqaaf sochiilee qaamaa ulfaataa armaan olitti ibsaman hojii keessan irratti sin qunnama? | Guyyaa: ______ |  |
| 119 | Guyyaatti yeroo hangamiif hojiilee sochii qaamaa ulfaata gaafatan kanniin hojjettu? | Guyyaa: ______ |  |
| 120 | Miillaan ykn biskileetiidhaan yoo xiqqaate daqiiqaa 10 fi isaa oliif walitti aansudhaan bakkaa bakkati ni sochootuu? | 1. Eeyyee 2. Lakki |  |
| 121 | Torbanitti guyyaa meeqaaf lukaan ykn biskileetiidhaan daqiiqaa 10 fi isaa oliif bakkaa bakkatti sochootu? | Guyyaa: ______ |  |
|  | **Waa’ee dhukkuba sukkaara** |  |  |
| 122 | Maatii kee keessa namni dhukkuba sukkaara qabu jira? | 1. Eeyyee 2. Lakki |  |
| 123 | Sukkaara gosa kami qabda | 1. Gosa tokkoffaa 2. Gosa lammaffaa |  |
| 124 | Yeroo hammamiif dhukkuba sukkaara waliin jiraatte? | ------------- |  |
| 125 | Yaala gosa kaam fudhatta? | 1.lilmoo  2. Kininii |  |
| 126 | Qorsa kee yeroon ni fudhatta? | 1. Eeyyee 2. Lakki |  |
| 127 | Safartuu sukkaara mana keessatti ni fayyadamta? | 1. Eeyyee 2. Lakki |  |
| 128 | Dhibeen sukkaraa dhiibbaa biraa sitti fide? | 1. Eeyyee 2. Lakki |  |
| 129 | Yoo eeyyen jette, maal sitti fide? | -------- |  |
| 130 | Gorsa doctoraa irratti hundoofte nyaata ni filattaa? | 1. Eeyyee 2. Lakki |  |
| 131 | Qorsa aadaa ni fayyadamta? | 1. Eeyyee 2. Lakki |  |
|  | **Nyaata** |  |  |
| 132 | Torban keessatti guyyoota meeqaaf muduraalee nyaatta? | Guyyaa: ______ |  |
| 133 | Torban keessatti guyyoota meeqaaf fuduraale nyaatta? | Guyyaa: ______ |  |
| 134 | Gosti zayitaa nyaata bilcheessuuf yeroo baay’ee mana keessatti itti fayyadamtan isa kami? | 1. Zayita atikiltii (jabaataa) 2. Zayita dhangala’aa 3. Dhadhaa 4. Margarini 5. Gonkumaa zayita hin fayyadamu 6. Hin beeku   Kan biroo:_________ |  |
| 135 | Giddu galeessaan torbanitti nyaata manatti hin qophoofne meeqa nyaatta? | Lakkoofsaan -------- |  |

Guca Gabaasa Laboraatori

| T.L | Maqaa laaboratorii | Bu’aa | yaada |
| --- | --- | --- | --- |
| 1. | Hemogiloobinii |  |  |
|  | Sukkaara |  |  |
|  | Dhiibbaa dhigaa |  |  |
|  | Hanqina dhiigaa | 1. Jira 2. Hin jiru |  |
| 5 | Gosa hanqina dhiigaa | -- |  |
|  | **Safartuulee hamma qaamaa** |  |  |
| 1. | **Hojjaa** |  |  |
| 2. | **Ulfaatina** |  |  |
| 3. | **BMI** |  |  |
